# 抗体偶联药物在晚期NSCLC中的临床进展和展望

**DOI:** 10.3779/j.issn.1009-3419.2025.102.29

**Published:** 2025-08-20

**Authors:** Yuling ZHONG, Jingyi WANG, Lin WU

**Affiliations:** ^1^410013 长沙，湖南省肿瘤医院/中南大学湘雅医学院附属肿瘤医院胸内二科（钟雨玲，王婧怡，邬麟）; ^1^The Second Department of Thoracic Oncology, Hunan Cancer Hospital/The Affiliated Cancer Hospital of Xiangya School of Medicine, Central South University, Changsha 410013, China; ^2^421001 衡阳，南华大学湖南省肿瘤医院研究生协作培养基地（钟雨玲）; ^2^Graduate Collaborative Training Base of Hunan Cancer Hospital, University of South China, Hengyang 421001, China

**Keywords:** 肺肿瘤, 抗体偶联药物, 生物标志物, 毒性反应, Lung neoplasms, Antibody-drug conjugates, Biomarker, Toxic reactions

## Abstract

靶向治疗和免疫治疗的进展已显著改善晚期非小细胞肺癌（non-small cell lung cancer, NSCLC）患者的临床结局，并重塑其治疗范式。然而，多数患者终将面临耐药问题，其后线治疗选择有限，疗效不尽如人意，成为当前临床实践的突出难点。近年来，抗体偶联药物（antibody-drug conjugates, ADCs）以其高效低毒的潜在优势，成为该领域关注的焦点。本文系统综述肺癌ADCs治疗领域的研究进展，并探讨当前面临的挑战。

## Abstract (en)

Advances in targeted therapy and immunotherapy have significantly improved clinical outcomes for patients with advanced non-small cell lung cancer (NSCLC), reshaping treatment paradigms. However, most patients ultimately face drug resistance, with limited options for subsequent therapies and suboptimal treatment efficacy, presenting a prominent challenge in current clinical practice. Antibody-drug conjugates (ADCs), characterized by high efficacy and favorable safety profiles, have emerged as a promising therapeutic frontier in recent years. This systematic review provides a comprehensive overview of the latest advancements in ADCs-based therapies for lung cancer, alongside discussions of the prevailing challenges in this rapidly evolving domain.

《2024年癌症统计报告》数据^[1]^显示，肺癌是中国乃至全球发病率和死亡率最高的恶性肿瘤。根据组织学类型分类，肺癌可分为非小细胞肺癌（non-small cell lung cancer, NSCLC）和小细胞肺癌（small cell lung cancer, SCLC），其中NSCLC约占85%^[2]^。近年来，尽管精准治疗在肺癌治疗领域取得了显著进展，靶向治疗与免疫治疗已逐渐成为晚期肺癌一线治疗的标准方案，显著改善了患者的预后并有效延长了生存期^[3]^，但获得性耐药和原发性耐药仍是不可避免的挑战。目前，因抗体药物偶联物（antibody-drug conjugates, ADCs）能够激发高效的抗肿瘤反应，同时显著减少对正常组织的毒性损害，其已成为肿瘤治疗研究的焦点，多种靶向人类表皮生长因子受体2（human epidermal growth factor receptor 2, HER2）、HER3、滋养层细胞表面抗原2（trophoblast cell-surface antigen 2, TROP-2）等不同抗原的ADCs正开发用于肺癌治疗^[4,5]^。本文将系统性梳理ADCs的结构和作用机制，综述热点研究并探讨现存问题。

## 1 ADCs的结构和作用机制

ADCs^[6]^主要由3个核心组成部分构成：特异性抗体、连接子以及细胞毒性药物。其特异性抗体能够高亲和力地靶向肿瘤细胞表面抗原，连接子则在维持抗体循环稳定性的同时精准调控药物释放，从而发挥高效的抗肿瘤活性，显著降低对正常细胞的毒性杀伤^[7,8]^。

### 1.1 ADCs的结构

#### 1.1.1 特异性抗体

在ADCs的设计中，理想的抗体应具备高特异性、高亲和力、长半衰期以及低免疫原性等关键特性。因此，人免疫球蛋白或人源化免疫球蛋白凭借其优异的生物特性而成为最常采用的抗体类型^[9]^。抗体作为糖蛋白分子，由抗原结合域和恒定片段两个关键结构组成，前者与抗原结合以精准识别癌细胞表面上的靶抗原，后者可通过抗体依赖的细胞介导的细胞毒作用（antibody-dependent cell-mediated cytotoxicity, ADCC）以及补体依赖的细胞毒作用（complement dependent cytotoxicity, CDC）来发挥免疫效应^[10]^。

#### 1.1.2 连接子

连接子作为ADCs的关键组成部分，通过化学键介导抗体与细胞毒性药物的定向偶联^[11]^，其结构设计直接决定了ADCs的药代动力学特性、靶标选择性释放效率及脱靶毒性阈值^[12]^。根据连接子的化学键断裂机制差异，分为可裂解和不可裂解两类^[13]^。可裂解连接子通过响应细胞内生理微环境差异，如pH值、谷胱甘肽浓度、蛋白酶或氧化还原电位等触发化学键断裂，其显著特点是不依赖溶酶体降解途径，可直接在胞质环境中完成细胞毒性药物释放。而不可裂解连接子则通过不可还原的化学键与抗体氨基酸残基形成牢固偶联，需经完整的内吞-溶酶体途径实现降解，此类型连接子因血浆稳定性显著提高，可有效降低脱靶毒性风险^[14]^。

#### 1.1.3 细胞毒性药物

细胞毒性药物（俗称“有效载荷”或“弹头”）相较于传统化疗药物具有显著的药理学优势，如nmol至pmol级的高效细胞杀伤效力、低分子量（通常<1 kDa）、优异的膜渗透性以及良好的化学稳定性^[15]^。常用的有效载荷包括微管蛋白抑制剂（如MMAE、DM1）、DNA损伤剂（如卡奇霉素、杜卡霉素）和拓扑异构酶抑制剂（如SN-38）^[16]^。此外，ADCs的细胞毒性强度及体内稳定性受药物抗体比（drug-to-antibody ratio, DAR）的显著影响^[17]^，即每个抗体分子偶联的药物分子数，其最优范围通常为4-8，以平衡效价与药代动力学特性。

### 1.2 ADCs的作用机制

ADCs的作用机制与靶抗原的选择密切相关^[18]^，其生物学效应包括诱导细胞凋亡、ADCC、CDC。首先，ADCs通过抗原-抗体特异性结合触发内化作用，使有效载荷靶向释放，直接诱导肿瘤细胞程序性死亡^[19,20]^。其次，ADCs抗体的Fc段可与效应细胞[如自然杀伤（natural killer, NK）细胞]表面的Fcγ受体结合，激活ADCC，促进免疫效应细胞对肿瘤细胞的杀伤。此外，抗体Fc段还能激活补体级联反应，形成膜攻击复合物，引发CDC，导致肿瘤细胞裂解。这种作用机制的有效性和安全性主要源于肿瘤相关抗原表达谱的异质性，包括抗原密度、内化效率及细胞表面定位等关键差异。

此外，ADCs除对靶抗原阳性肿瘤细胞产生直接抗肿瘤效应外，还可通过“旁观者效应”^[21]^（或“旁观者杀伤”）介导对邻近非靶抗原肿瘤细胞的杀伤。其机制涉及：ADCs内化后释放的有效载荷通过跨膜渗透扩散至邻近细胞；肿瘤微环境的酸性条件诱导可裂解连接子裂解，促使部分有效载荷胞外释放；同时，ADCs诱导的免疫原性细胞死亡可激活局部免疫应答。该效应不仅能克服肿瘤的异质性，增强治疗的强度和广度，还能减少耐药的风险。

## 2 ADCs在NSCLC中的研究进展

在当前的肺癌研究领域，最受关注的ADCs靶点主要包括：HER2、HER3、TROP-2以及间质-上皮转化因子（mesenchymal-epithelial transition factor, MET）等（详见表1^[22-35]^）。

**表1:** ADCs在晚期NSCLC中的临床研究汇总

ADCs	Target	Trial	Setting	Treatment	Efficiency	ADEs (≥grade 3)	
						Proportion	Major DRA
T-DXd	HER2	DESTINY-Lung01^[22,23]^	Metastatic *HER2*-mutant NSCLC	T-DXd (6.4 mg/kg)	ORR: 55%; mPFS: 8.2 mon; mOS: 17.8 mon	46.0%	Neutropenia: 19%
		DESTINY-Lung02^[24,25]^	Previously treated patients with *HER2* metastatic NSCLC	T-DXd (6.4 mg/kg）	ORR: 56%; mPFS: 12.9 mon; mOS: 17.3 mon	60.0%	-
				T-DXd (5.4 mg/kg）	ORR: 50%; mPFS: 10.0 mon; mOS: 19.0 mon	39.6%	-
		DESTINY-Lung05^[26]^	Chinese patients with *HER2* metastatic non-squamous NSCLC	T-DXd (5.4 mg/kg）	ORR: 58.3%; mPFS: NE; 12-yr PFS: 55.1%	51.4%	Neutropenia: 26.4%
HER3-DXd	HER3	HERTHENA-Lung01^[27]^	Previously treated patients with advanced *EGFR*-mutated NSCLC	HER3-DXd	ORR: 29.8%; mPFS: 5.5 mon; mOS: 11.9 mon	64.9%	Hematologic toxicities (-)
		HERTHENA-Lung02^[28]^	Advanced *EGFR* mutant (Ex19del or L858R) NSCLC	HER3-DXd *vs* PBC	ORR: 35.2% *vs* 25.3%; mPFS: 5.8 *vs* 5.4 mon; mDOR: 5.7 *vs* 5.4 mon	73% *vs* 57%	Thrombocytopenia: 30% *vs* 7.9%
Dato-DXd	TROP2	TROPION-Lung01^[29]^	Previously treated advanced NSCLC	Dato-DXd *vs* Docetaxel	mPFS: 4.4 *vs* 3.7 mon; mOS: 12.9 *vs* 11.8 mon	25.6% *vs* 42.1%	-
		TROPION-Lung05^[30]^	Advanced NSCLC with AGA	Dato-DXd	ORR: 35.8%	28.5%	Stomatitis: 9.5%
		TROPION-Lung02^[31]^	Advanced NSCLC	Dato-DXd plus Pembrolizuma *vs* Dato-DXd plus Pembrolizuma with Pt-CT	ORR: 55.0% *vs* 56.0%; mPFS: 11.2 *vs* 6.5 mon	-	-
		TROPION-Lung04^[32]^	Advanced NSCLC	Dato-DXd+Durvalumab vs Dato-DXd+Durvalumab+Carboplatin	ORR: 50.0% *vs* 76.9%	42.1% *vs* 71.4%	Stomatitis: 10.5% *vs* 7.1%
Sac-TMT	TROP2	OptiTROP-Lung03^[33,34]^	Previously treated patients with advanced *EGFR*-mutated NSCLC	Sac-TMT *vs* Docetaxel	ORR: 45.1% *vs* 15.6%; mPFS: 6.9 *vs* 2.8 mon	56.0% *vs* 71.7%	Neutropenia: 42.9% *vs* 58.7%
Teliso-V	c-MET	LUMINOSITY^[35]^	Previously treated c-Met protein-overexpressing advanced nonsquamous NSCLC	Teliso-V	ORR: 28.6%; mPFS: 5.7 mon; mOS: 14.5 mon	56.4%	Peripheral sensory neuropathy: 7%

ADCs: antibody-drug conjugates; ADEs: adverse drug events; T-DXd: Trastuzumab deruxtecan; NSCLC: non-small cell lung cancer; ORR: objective response rate; OS: overall survival; PFS: progression-free survival; HER3-DXd: Patritumab deruxtecan; NE: not evaluated; Dato-DXd: Datopotamab deruxtecan; AGA: actionable genomic alterations; Pt-CT: platinum chemotherapy; PBC: platinum-based chemotherapy; Sac-TMT: Sacituzumab tirumotecan; Teliso-V: Telisotuzumab vedotin; HER2: human epidermal growth factor receptor 2; EGFR: epidermal growth factor receptor; DOR: duration of response.

### 2.1 靶向HER2的ADCs

德曲妥珠单抗（Trastuzumab deruxtecan, T-DXd）是一种新型ADC药物，通过可裂解的四肽连接子将人源化的抗HER2单克隆抗体曲妥珠单抗与拓扑异构酶I抑制剂DXd偶联而成。T-DXd由于其高DAR（约8）和高膜通透性载荷，可诱导细胞毒作用，从而实现旁观者效应。

DESTINY-Lung01研究^[22,23]^旨在评估T-DXd治疗（6.4 mg/kg）既往治疗失败的HER2基因过表达/突变晚期NSCLC的有效性和安全性。研究结果显示，55%的患者实现了客观缓解率（objective response rate, ORR）的显著提高，中位无进展生存期（median progression-free survival, mPFS）可达8.2个月，中位总生存期（median overall survival, mOS）延长至17.8个月，最常见的药物不良反应（adverse drug event, ADE）为中性粒细胞减少。2022年欧洲肿瘤内科学会（European Society for Medical Oncology, ESMO）公布了T-DXd治疗HER2过表达晚期NSCLC队列的研究结果^[36]^。数据显示，HER2过表达组的ORR为26.5%，mPFS和mOS分别为5.7和12.4个月，其中主要ADEs为恶心与食欲下降。同时，DESTINY-Lung03研究^[37]^也证实了T-DXd对HER2过表达晚期非鳞NSCLC的显著疗效与可控安全性。随后DESTINY-Lung02研究^[24,25]^则进一步探索了T-Dxd不同剂量（5.4和6.4 mg/kg）对HER2突变难治性/复发性NSCLC的疗效。值得注意的是，两组不同剂量在疗效（ORR：50.0% *vs* 56.0%，mPFS：10.0 *vs* 12.9个月，mOS：19.0 *vs* 17.3个月）以及ADE耐受（≥3级ADE：39.6% *vs* 60.0%）方面，均可带来显著的临床获益和安全可控。DESTINY-Lung05研究^[26]^在中国人群中展现的积极疗效，也为该患者群体的治疗决策增添关键循证依据。基于以上研究中T-DXd展现出的高效性和低毒性，美国食品药品监督管理局（Food and Drug Administration, FDA）批准德曲妥珠单抗（5.4 mg/kg）在标准治疗失败HER2突变NSCLC中的适应证，使其成为全球首个在肺癌领域获批的ADC。此外，III期DESTINY-Lung04/06研究正进一步探索T-DXd对比一线免疫联合化疗用于HER2突变/过表达晚期非鳞状NSCLC患者的疗效与安全性，旨在为初治患者提供新的治疗方案。DESTINY-Lung系列研究正在积极进行，涵盖不同治疗剂量、方案及治疗线数等关键领域，并期待T-DXd在进一步的III期临床试验中取得重大进展。

同时，国产HER2 ADCs也取得了突破性进展。由中国学者牵头开展的HORIZON-Lung研究^[38]^是一项多中心、单臂II期临床试验，旨在评估新型HER2靶向ADC瑞康曲妥珠单抗（Trastuzumab rezecan, SHR-A1811）在经治的晚期HER2突变NSCLC患者中的疗效与安全性。该研究结果显示，SHR-A1811治疗组的ORR达73.4%，mPFS延长至11.5个月，且在脑转移亚组（ORR为87.5%）和HER2-酪氨酸激酶抑制剂（tyrosine kinase inhibitors, TKIs）经治患者（ORR: 77.3%）中均观察到显著获益；治疗相关间质性肺炎发生率为7%，药物安全性特征优异。基于此项研究结果，国家药品监督管理局（National Medical Products Administration, NMPA）已批准SHR-A1811用于HER2突变晚期NSCLC的二线及后线治疗。此外，目前关于HER2 ADCs正在如火如荼地研究中，如RC48、A166等，其疗效与安全性有待前瞻性大型临床试验进一步验证。

### 2.2 靶向HER3的ADCs

有研究^[39-41]^表明，表皮生长因子受体（epidermal growth factor receptor, EGFR）-TKIs抑制EGFR激酶活性后，通过上调HER3表达并促进其与EGFR/HER2形成异源二聚体，激活下游PI3K/Akt和MAPK通路，最终增强肿瘤细胞存活及侵袭能力，这提示靶向HER3可能成为克服EGFR-TKIs耐药的关键策略。

I期HERTHENA-Lung01研究^[42]^显示，德帕瑞妥单抗（Patritumab deruxtecan, HER3-DXd）针对既往接受EGFR-TKIs及标准化疗后进展的EGFR突变NSCLC患者展现出抗肿瘤活性且安全性可控，因此FDA授予其突破性疗法称号，并推动后续深入研究开展。2023年世界肺癌大会报道了II期HERTHENA-Lung01研究结果^[27]^，在225例可评估患者中，HER3-DXd的ORR为29.8%，PFS为5.5个月，OS为11.9个月。值得注意的是，包括脑转移状态、HER3表达水平及EGFR-TKIs耐药机制在内的各亚组患者均观察到临床获益。此外，既往接受过奥希替尼（Osimertinib）和化疗的亚组患者也显示出相似的生存获益。随后多中心、随机的III期研究HERTHENA-Lung02结果^[28]^表明，尽管HER3-DXd相较于传统化疗PFS延长了0.4个月且具有统计学意义，然而OS生存曲线出现了交叉现象，这表明HER3-DXd未能为该患者群体带来显著的生存获益。因此，HER3虽作为EGFR-TKIs耐药后极具潜力的治疗靶点，但其疗效是否优于现有方案、ADE能否得到有效管理，以及最终能否改变当前治疗格局，仍需大型随机对照试验进一步验证。

### 2.3 靶向TROP2的ADCs

人TROP2广泛表达于肺、乳腺、胃等上皮组织，且在肿瘤组织中显著高表达^[43,44]^；其在肺腺癌（64%）和鳞癌（75%）中的高表达尤为突出^[45,46]^。基于TROP2在肿瘤细胞中内吞活性显著高于正常组织的生物学特性^[47]^，该靶点已成为肺癌ADCs的优选靶标。TROPION-Lung01研究^[29]^作为该领域中首个报道PFS阳性结果的III期临床试验，标志着ADCs应用的新开端。研究结果显示，与多西他赛（Docetaxel）相比，德达博妥单抗（Datopotamab deruxtecan, Dato-DXd）在伴有可靶向基因组改变（actionable genomic alterations, AGAs）的经治非鳞状NSCLC患者中PFS和OS分别延长了1.9个月（5.5 *vs* 3.6个月；HR=0.63）和2.3个月（14.6 *vs* 12.3个月；HR=0.84），且≥3级治疗相关ADEs发生率仅为25.6%。此外，TROPION-Lung05研究^[30]^显示，Dato-DXd在EGFR突变经治亚组中ORR达43.6%，显著高于总人群的35.8%，提示其对EGFR突变亚组疗效突出。随后TROPION-Lung01及TROPION-Lung05研究的汇总分析^[48]^则进一步扩大样本量，阳性结果为Dato-DXd在EGFR突变晚期NSCLC后线治疗领域的应用提供了强有力的支持。目前，Dato-DXd单药在既往接受治疗的局部晚期或转移性NSCLC患者中展现的显著抗肿瘤疗效，推动了其联合治疗模式的深入探索。TROPION-Lung02研究^[31]^首次评估了Dato-DXd联合帕博利珠单抗（Pembrolizumab）（双联）±化疗（三联）在初治晚期NSCLC中的疗效，研究结果显示，双联方案[ORR：55%，疾病控制率（disease control rate, DCR）：88%，mPFS：11.2个月]和三联方案（ORR：56%，DCR：89%，mPFS：6.8个月）均显示出临床获益。TROPION-Lung04^[32]^研究进一步证实，在未经免疫治疗的晚期/转移性NSCLC患者（含初治、放疗后进展及一线化疗经治人群）中，Dato-DXd联合度伐利尤单抗（Durvalumab）的ORR和DCR分别为50.0%、92.9%；而联合度伐利尤单抗及化疗的三联方案ORR提升至76.9%，DCR达92.3%，且所有程序性细胞死亡配体1（programmed cell death ligand 1, PD-L1）亚组均观察到缓解，两项研究^[31,32]^均提示三联疗法ORR更优。三联组ORR优势可能源于化疗的快速缩瘤作用，双联组mPFS优势则提示免疫与ADCs协同的持久获益潜力，这可能说明临床治疗需平衡快速缓解与长期控制的不同治疗目标。此外，正在开展的TROPION-Lung07^[49]^、TROPION-Lung08^[50]^及AVANZAR^[51]^研究，均旨在深入探索并精准评估TROP2 ADCs联合治疗在NSCLC一线治疗中的疗效与安全性。其中，TROPION-Lung07研究^[49]^针对PD-L1肿瘤细胞阳性比例分数（tumor proportion score, TPS）<50%的晚期或转移性NSCLC，探索Dato-DXd联合帕博利珠单抗±化疗的疗效；TROPION-Lung08研究^[50]^进一步比较Dato-DXd联合帕博利珠单抗与帕博利珠单抗单药在驱动基因阴性且PD-L1 TPS≥50%的晚期或转移性NSCLC一线治疗中的差异；AVANZAR研究^[51]^则评估Dato-DXd联合度伐利尤单抗及卡铂作为一线方案治疗局部晚期/转移性NSCLC的疗效及安全性。

芦康沙妥珠单抗（Sacituzumab tirumotecan, Sac-TMT）作为全球首个获批肺癌领域的靶向TROP2的ADC，填补了经治EGFR突变晚期NSCLC患者的治疗空白，标志着中国国产药物在该领域的标志性突破。OptiTROP-Lung03研究^[33,34]^是一项多中心、随机对照II期临床试验，旨在比较Sac-TMT与多西他赛在既往接受过EGFR-TKIs和标准化疗失败的晚期EGFR突变NSCLC患者中的疗效和安全性。研究结果显示，与多西他赛相比，Sac-TMT组ORR显著提高了29.5%（ORR: 45.1% *vs* 15.6%; *P*<0.001），mPFS延长了4.1个月（mPFS：6.9 *vs* 2.8个月；HR=0.30；*P*<0.001），尽管两组均未达到mOS，但Sac-TMT组已显示出显著的OS改善趋势，死亡风险降低了51%（HR=0.49, P=0.007）。基于OptiTROP-Lung03研究取得的积极结果，Sac-TMT在EGFR突变晚期NSCLC的治疗策略正向一线延伸，并加速从单药向联合方案的拓展。

### 2.4 靶向c-MET的ADCs

MET过表达（30%-50%）、MET 14号外显子跳跃突变（3%）及MET扩增（1.5%）均为NSCLC明确的驱动因素^[52-54]^。其中，针对MET 14号外显子跳跃突变患者，卡马替尼（Capmatinib）、特泊替尼（Tepotinib）等TKIs已获FDA批准；而对于MET过表达和扩增，目前尚无获批的靶向治疗方案。

特立妥珠单抗（Telisotuzumab vedotin, Teliso-V）^[55]^由人源化单克隆抗体Telisotuzumab组成，通过可裂解的缬氨酸-瓜氨酸连接物偶联微管抑制剂Monomethyl auristatin E，DAR为3.1。II期LUMINOSITY研究^[35]^评估了Teliso-V对c-Met过表达、EGFR野生型非鳞状NSCLC的疗效。结果显示，172例可评估患者的总体ORR为28.6%，其中c-Met高表达亚组ORR可达34.6%，中表达亚组为22.9%；mOS为14.5个月，高、中表达亚组分别为14.6和14.2个月；mPFS为5.7个月，高、中表达亚组分别为5.5和6.0个月，最常见ADE为周围感觉神经病变。这暗示着Teliso-V在c-Met过表达非鳞状EGFR野生型NSCLC中显示出持久抗肿瘤活性，尤其在c-Met高表达患者中疗效更优，且ADEs总体可控，该药已成为全球首款获批的c-Met ADC。目前，III期TeliMET NSCLC-01试验正针对Teliso-V开展进一步评估，旨在为经治c-Met过表达NSCLC患者提供循证医学证据并确立新治疗标准。

### 2.5 靶向其他靶点的ADCs

酪氨酸蛋白激酶7（protein tyrosine kinase 7, PTK7）^[56,57]^、粘连蛋白4（Nectin 4）^[58]^、组织因子（tissue factor, TF）^[59]^、人癌胚抗原相关的细胞黏附分子5（carcinoembryonic antigen-related cell adhesion molecule 5, CEACAM5）^[60]^等靶向其他靶点的ADCs临床研究正在如火如荼地进行中。这些研究不仅展示了ADCs在抗肿瘤治疗中的潜力，而且已经初步显示出一定的抗肿瘤活性。随着研究的深入，我们期待未来能够获得更进一步的临床研究结果，以验证ADCs在真实世界应用中的有效性和安全性。

## 3 ADCs治疗的挑战和思考

### 3.1 药物疗效和毒性

尽管针对HER2、HER3、TROP2和c-MET等不同靶点的ADCs在晚期NSCLC中均显示出抗肿瘤活性，但靶点表达水平、靶向位点、药物结构以及DAR等差异均可显著影响其疗效和安全性。作为新型抗肿瘤药物，ADCs因连接子不稳定导致有效载荷过早释放，或特异性抗体与正常细胞抗原结合等因素导致的ADEs^[61]^不容忽视。其中，以贫血、中性粒细胞减少症、血小板减少症等血液毒性最为常见。值得注意的是，一项荟萃分析^[62]^显示，基于MMAE有效载荷的ADCs主要导致血液学毒性，如中性粒细胞减少、血小板减少发生率分别为37.0%和28.8%，而DM1有效载荷则与肝毒性和周围神经病变强相关。靶点特异性分析发现，抗HER2 ADCs的肝酶异常发生率显著高于其他靶点（*P*<0.01），而CD30靶向药物更易引发≥3级中性粒细胞减少（发生率：72.9% *vs* 其他靶点均值46.1%）。上述数据表明，ADCs的毒性特征存在显著结构-效应关系，这为临床药物选择及毒性监控提供了分子层面的理论支撑，并强调了根据ADCs的载荷类型制定个性化ADEs管理策略的必要性。此外，在DESTINY-Lung02试验^[24]^中，接受最高剂量T-Dxd治疗的患者ADEs发生率约为低剂量组的2倍，这提示ADEs似乎与药物剂量存在相关性。而间质性肺疾病（interstitial lung disease, ILD）是ADCs使用期间最严重且影响生活质量的毒性反应。9项I和II期T-DXd单药治疗研究的汇总分析^[63]^显示，ILD的总发生率为15.4%，87.0%患者为≤2级ILD，5级ILD发生率为2.2%。综上，ADCs的ADEs类型和程度具有显著异质性，其发生机制与剂量、有效载荷及分子结构等多维度因素密切相关，因此需重点关注关键要素以优化临床应用安全性，同时也期待更多大型III期临床研究的探索。

### 3.2 生物标志物

当前肺癌研究领域，针对ADCs疗效及预后相关的生物标志物在临床试验探索性分析中仍处于初级阶段，现有研究主要聚焦于靶抗原表达水平。DESTINY-Lung01关键性研究^[36]^显示，低剂量T-DXd对HER2突变免疫组化（immunohistochemistry, IHC）3+患者的ORR可达52.9%，但IHC 2+组仍存在可观的临床获益（ORR: 20.8%），这提示单一靶蛋白的标志物存在明显的预测局限性。LUMINOSITY研究^[35]^的亚组分析也得出了类似结论，在172例非鳞状EGFR野生型NSCLC患者中，c-Met表达水平的高低与疗效之间并非简单的线性关系，而是受到耐药机制、肿瘤微环境、共表达分子等多种因素的影响。上述结果表明，免疫组化评分阈值不统一及肿瘤异质性带来的技术挑战，进一步限制了生物标志物的研究进展。但值得注意的是，在对TROPION-Lung01进行回顾性探索性分析^[64]^时，研究人员通过定量连续评分（quantitative continuous scoring, QCR）计算TROP2等靶点的标准化膜比例（normalized membrane ratio, NMR）可精确预测ADCs的临床疗效。结果显示，与TROP2 QCS-NMR阴性组相比，TROP2 QCS-NMR阳性组ORR更高（55.0% *vs* 27.8%）及mPFS更长（9.6 *vs* 5.7个月；HR=0.46）。这一发现的深入探索将为ADCs生物标志物研究及精准治疗开辟新路径，未来需突破传统IHC二分法局限，超越单一膜蛋白表达或基因突变等单一维度，以拓展更广泛的精准识别策略。此外，更深层次的问题在于，ADCs作用机制的关键环节错综复杂以及治疗过程中动态变化的分子特征，现有探索性分析普遍缺乏系统的多组学整合^[65,66]^。针对这些瓶颈，未来研究需要构建更全面的预测模型，整合包括基线多组学特征、动态监测指标和功能性验证在内的多维数据体系。

## 4 小结与展望

当前，ADCs在肺癌后线治疗领域已取得突破性疗效，并逐步向一线治疗领域延伸；同时，为进一步优化患者生存获益，多项研究围绕靶点选择、联合策略及分子结构优化等方向展开了深入探索。然而，新药应用亦带来不容忽视的新问题，如毒性反应的处理、生物标志物的确定等，这需要更加精细和个体化的方案，以确保患者的安全和疗效的最大化。我们相信，随着研究的不断深入，ADCs将在肺癌领域展现出令人鼓舞的治疗前景。
